# The hip joint mobilization with movement technique improves muscle activity, postural stability, functional and dynamic balance in hemiplegia secondary to chronic stroke: a blinded randomized controlled trial

**DOI:** 10.1186/s12883-023-03315-2

**Published:** 2023-07-11

**Authors:** Soudeh Arabzadeh, Fahimeh Kamali, Soha Bervis, Mohsen Razeghi

**Affiliations:** 1 Student Research Committee, School of Rehabilitation Sciences, Shiraz University of Medical Sciences, Shiraz, Iran; 2 Physical Therapy Department, School of Rehabilitation Sciences, Shiraz University of Medical Sciences, Shiraz, Iran; 3 Rehabilitation Sciences Research Center, Shiraz University of Medical Sciences, Shiraz, Iran

**Keywords:** Stroke Rehabilitation, Stroke, Hip, Balance, EMG

## Abstract

**Background:**

People with stroke generally experience abnormal muscle activity and develop balance disorder. Based on the important role of the proximal joints of the lower extremity in balance maintenance, hip joint mobilization with movement technique can be applied to enhance normal joint arthrokinematics. Therefore, the present study aimed to investigate the effectiveness of hip joint mobilization with movement technique on stroke patients’ muscle activity and balance.

**Methods:**

Twenty patients aged between 35 and 65 years old with chronic stroke were randomly assigned either to an experimental group (n = 10) or to a control group (n = 10). Both groups participated in a 30-minute conventional physiotherapy session 3 times per week for 4 weeks. The experimental group received an additional 30-minute’s session of hip joint mobilization with movement technique on the affected limb. The muscle activity, berg balance scale, time up and go, and postural stability were measured at baseline, 1-day and 2-week follow-up by a blinded assessor.

**Results:**

The experimental group showed a significant improvement in berg balance scale, time up and go, and postural stability (p ≤ 0.05). The rectus femoris, tibialis anterior, biceps femoris, and medial gastrocnemius muscles’ activations of the affected limb during static balance test markedly changed along with the biceps femoris, erector spine, rectus femoris, and tibialis anterior muscles during dynamic balance test after hip joint mobilization with movement technique. The mean onset time of rectus abdominus, erector Spine, rectus femoris, and tibialis anterior muscles activity significantly decreased in the affected limb after hip joint mobilization with movement technique compared to the control group (p ≤ 0.05).

**Conclusions:**

The results of the present study suggest that a combination of hip joint mobilization with movement technique and conventional physiotherapy could improve muscle activity and balance among chronic stroke patients.

**Trial registration number:**

The study was registered in the Iranian Registry of Clinical Trials (No; IRCT20200613047759N1). Registration date: 2/08/2020.

## Background

Balance as an essential motor performance provides the foundation for functional tasks and is negatively affected by motor control disorders, including muscle incoordination and weakness. The delayed onset of agonist muscle recruitment and changes in muscle timing and sequencing affect the efficiency of energy generation in postural muscles at the appropriate speed against perturbations during movements and functional tasks [1–3]. As a result, abnormal muscle activity and balance disorders frequently present in post-stroke patients.

According to the recent findings, selective motor control may be more important in balance control in the proximal part of the lower extremity than in the distal part [4, 5]. Hip disorders are attributed to impaired both static and dynamic balance following the lack of interplay among various mechanisms such as sensory afferents, motor control, and adequate joint movement [6, 7]. The hip joint strategy is predominant in stroke, playing an effective role in the postural correction by creating hip joint torque [8]. It has been proposed that pelvic stability of post-stroke patients is influenced by hip muscles weakness to weight bearing asymmetry during standing and gait [9, 10]. The proximal dynamic stability of pelvic depends on the coordination between lower trunk and hip muscles activity due to hip muscle attachment to the pelvic bone and lumbar spine [11]. The importance of the hip joint in standing balance control needs further investigations in this population.

Joint mobilization techniques are broadly used for orthopedic rehabilitation, such as hip joint, to improve the abnormal muscle tone as well as muscle length and joint mobility, which could return higher levels of activities of daily living [12–14]. Mulligan proposed a combination of joint gliding techniques with active or passive osteokinematic (physiological or angular) motion, called mobilization with movement technique [15]. Besides peripheral and central effects, mobilization with movement technique can improve joint arthrokinematics and motor control by soft tissue stretching [16].

Despite the widespread use of mobilization with movement technique for peripheral joint dysfunction in musculoskeletal physiotherapy, its efficacy in electromyography has not been studied [17]. To the best of our knowledge, no study has investigated the effectiveness of hip joint mobilization with movement technique in combination with conventional physiotherapy in post-stroke patients. Hence, this study investigated the effects of hip joint mobilization with movement technique on the activity of lower extremity and trunk muscles, postural control, and functional and dynamic balance in post-stroke patients, 1-day and 2-weeks after the treatment.

## Materials and methods

### Trial design

**Fig. 1:**
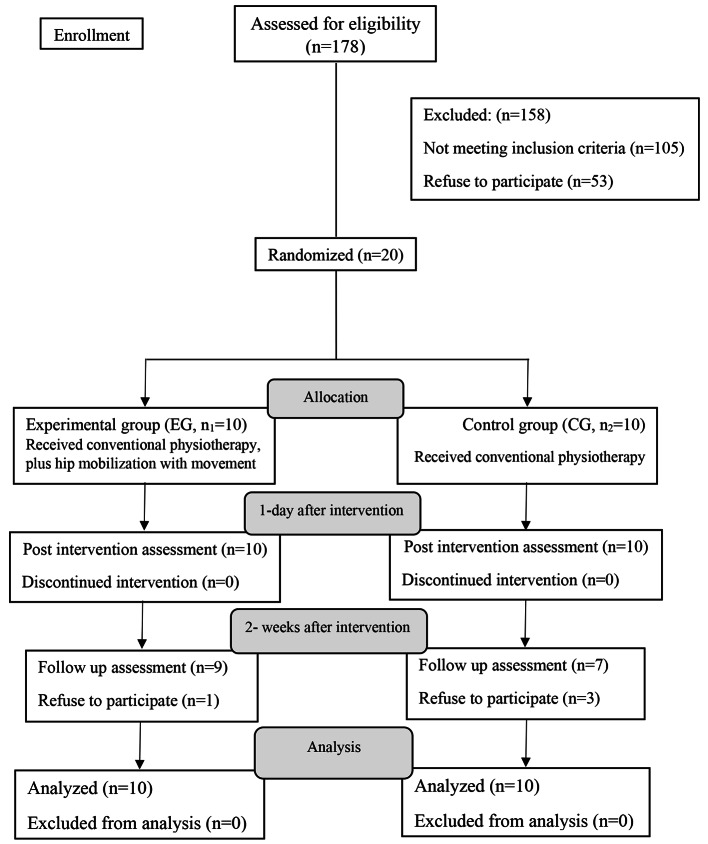
Flow chart of the study

This single-blind randomized controlled trial was conducted with 2 parallel groups study at the physiotherapy clinic and biomechanical laboratory of the school of Rehabilitation Sciences of Shiraz University of Medical Sciences, from August 2020 to July 2021.The inclusion criteria were hemiplegia secondary to stroke > 6 months and < 18 months, ages ranging from 35 to 65 years, the ability to follow verbal commands, the ability to walk independently, a score ≥ 24 in the Persian Mini-Mental State Examination scale [18], and Brunnstrom stages of stroke recovery 2–4. The exclusion criteria included neurological and orthopedic conditions, visual or auditory impairments, stroke in cerebellum, contraindications for hip mobilization, and surface electromyography (cancer, having pacemaker, unstable epilepsy, or skin abnormalities). A written and oral explanation about the study was given to all eligible participants. The 20 subjects voluntarily signed a written informed consent form before enrollment (Fig. 1). The approval of the Ethics Committee of Shiraz University of Medical Sciences, Shiraz, Iran (No: 98-01-06-21446) was concurrently obtained and the study was registered in the Iranian Registry of Clinical Trials (No; IRCT20200613047759N1).

### Sample size

A pilot sample size of 20 participants (to cover a possible dropout of 25%) was conducted by considering the post-treatment Medial-Lateral dynamic stability index as the main variable. METCALC® software (Ver. 5.43; Technische University Wien) was used to achieve a power of at least 0.8 at a significant level of α = 0.05, and an effect size of 1.49. Finally, 14 subjects (7 subjects per group) were recruited to suffice the target power.

### Assessments

#### Postural stability

Postural stability indices were evaluated using a Biodex Balance System SD (BBS, Shirley, NY, USA) with a circular platform that tilts up to 20° in a 360° direction. The platform stability was varied from 1 (least stable) to 12 (most stable). The angular excursion of the center of gravity was calculated in terms of overall stability, and Medial-Lateral (ML), and Anterior–Posterior (AP) stability indices [19, 20]. A high score is indicative of a lot of motion and trouble balancing, so a lower score is more desirable than a higher score. The postural control protocol consisted of two different conditions, for static condition, subject stands in bare feet and arms next to the body on a locked platform and for dynamic condition, a movable platform was set at level 10 [21]. Each trial was performed for 20 s in triplicate, with the mean value calculated.

#### Berg balance scale

A valid and reliable Persian version of the berg balance scale was used to assess the participants’ functional balance through fourteen separate motor skill items rated on a 5-point scale ranging from 0 (needing maximum help) to 4 (independent) [22]. The total score of the scale was 56.

#### Timed up and go test

Timed up and go test was the time measured by a stopwatch when the patient raised from a chair, walked 3 m, turned around, walked back, and then sat down with the maximum effort to measure the dynamic balance ability. The mean value of the three measurements was calculated as the final score. A 2-minutes rest period was considered between each two trials. Excellent inter-rater (ICC = 0.98) and intra-rater reliability (ICC = 0.99) values have been reported for this test [23, 24].

#### Muscle activity pattern

Surface electromyography (Biometrics Ltd, Nine Mile Point Ind. Est. UK) was bilaterally recorded during static and dynamic balance tests, using Biodex Balance System, in terms of SENIAM recommendations for rectus abdominus (2 cm lateral to the umbilicus), erector spine (2 fingers width lateral to the L3 spinous processes), rectus femoris (at 50% on the line from the anterior superior iliac spine to the superior part of the patella), biceps femoris (at 50% on the line between the ischial tuberosity and the lateral epicondyle of the tibia), tibialis anterior (one third of the distance between the tip of the fibula and the tip of the medial malleolus), and medial gastrocnemius (on the most prominent bulge of the muscle) [25, 26].

The myoelectric signals were recorded with a 16-channel electromyography device at the sampling rate of 1000 HZ and then processed with 50 HZ notch filter and 20 to 450 Hz band pass filter. Filtration was applied post-recording using MATLAB® software (the MathWorks, Inc., Ver. 2015a, USA). The ground electrode was placed over the lateral malleolus using a conductive gel. Pairs of Ag/AgCl bipolar surface electrodes were fixed along the long axis of the muscles with 2 cm center-to-center distance [27]. Skin shaving and cleaning were performed with 70% alcohol before electrode placement. The sensor was attached with a double-sided sticky tape and an anti-allergy tape for better bonding.

Raw electromyography (EMG) data were full-wave rectified, and then the root mean square was calculated for each trial. The onset time was considered while the values, in millivolts, exceeded the mean level of the baseline activity by two standard deviations for a minimum of 30 ms. To normalize the EMG data, the root mean square of reference voluntary contraction was determined using the following methods: the middle three seconds of trunk reference voluntary contraction, and the five seconds of lower extremity reference voluntary contraction with no resistance: 5-sec supine isometric trunk curl-up, 5-sec prone isometric trunk extension for rectus abdominus and erector spine respectively, and 10-sec upright standing posture for rectus femoris, biceps femoris, tibialis anterior, and medial gastrocnemius. Data were averaged over three trials and then reported as reference voluntary contraction % for both groups.

### Experimental procedures

The participants were randomly assigned either to the experimental group or to the control group using the permuted block randomization technique with equal allocation (block size of 4). Random allocation and statistical analysis were performed by a statistician who was blinded to the grouping. Both groups received 30 min conventional physiotherapy, including active and passive range of motion (ROM), weight bearing exercises, balance and gait training, three sessions per week for four weeks. The experimental group received an additional 30 min of hip joint mobilization with movement. The outcome measures were evaluated at baseline, 1-day and 2-weeks after the treatment by an experienced physiotherapist who was not familiar with the groups’ allocation.

Hip joint mobilization with movement technique was applied to the affected limb, as described by Mulligan [28], by a physiotherapist familiar with the technique as follows:

Internal-external rotation: The patient was lying in a supine position, with the hip and knee bent. The Mulligan belt was placed around the patient’s upper thigh and just below the therapist’s hip joints. The patient actively rotated the hip while the therapist applied lateral glide [28].

Flexion: The basic parts were similar to those of the rotation technique. While the therapist applied lateral glide, the patient actively flexed his thigh [28].

Abduction-adduction: While the patient was in a supine position, the therapist applied longitudinal traction to the thigh through the belt wrapped around the therapist’s arms. The patient actively abducted and adducted the thigh while maintaining longitudinal traction [28].

A grade III glide was sustained for 10 s in 3 sets of 6 repetitions with a 1-minute break between sets and a 5-second rest between repetitions [28].

### Statistical analysis

IBM® SPSS Statistics for Windows, version 25.0. Armonk, NY: IBM Corp was used for statistical analyses. Shapiro-Wilk test was performed to examine data distribution. Baseline variables were compared between groups using the independent t-test for continuous data and the chi-square test for categorical data. Continuous variables were reported as the mean and standard deviation. The categorical variables were stated as frequencies. A 2 × 3 (group × time) repeated measure ANOVA was performed to compare within- and between-group differences. The time variables were baseline, 1-day and 2-weeks after treatment, and the group variables were experimental and control group. Mann-Whitney U-test was used to compare between-group means on the one day and two weeks after the interventions. The Bonferroni post hoc test was performed for both groups to determine the effect of intervention in each time periods. Statistical significance level was set at p < 0.05.

## Results

A total of twenty stroke patients (10 patients in each group) were recruited to participate in the current study. While four dropouts were recorded during the follow-up assessment, other participants completed the study protocol. No significant differences were observed between groups (P > 0.05) in baseline characteristics and physical parameters (Table 1).

**Table 1:** Subjects characteristics

Variables	Group		P_V_
	Experimental group (n = 10)	Control group (n = 10)	
Sex			
Male Female	6 4	7 3	0.18^†^
Side of hemiplegia			
Left Right	4 6	3 7	0.65^†^
Type of stroke (n)			
Hemorrhagic Ischemic	4 6	4 6	0.37^†^
Age (years)	55.60 ± 7.18	56.10 ± 9.19	0.89^§^
Height (cm)	167 ± 12.18	165 ± 12.12	0.71^§^
Weight (kg)	74.97 ± 14.97	81.26 ± 15.29	0.36^§^
Time since stroke (months)	10.30 ± 3.62	12.87 ± 4.32	0.28^§^
Pre TUG	52.72 ± 46.16	51.93 ± 43.14	0.94^‡^
Pre BBS	41.50 ± 4.03	41 ± 2.78	0.54^‡^
Overall-static	2.25 ± 1.75	2.17 ± 1.95	0.59^‡^
Overall-dynamic	1.95 ± 1.24	2.09 ± 1.44	0.76^‡^
AP static	1.37 ± 1.02	1.52 ± 1.42	0.70^‡^
AP dynamic	1.15 ± 0.51	1.01 ± 1.13	0.09^‡^
ML static	1.57 ± 1.34	1.32 ± 1.28	0.62^‡^
ML dynamic	1.31 ± 0.84	1.65 ± 1.02	0.51^‡^

Values are expressed as mean ± standard deviation or frequency.

Abbreviation: BBS, berg balance scale; TUG, time up and go test; AP, Antroposterior; ML, Medio lateral.

§ Independent student t test.

† Chi- squared test.

‡ Mann-Whitney U-test.

Repeated measures ANOVA showed a significant reduction in the timed up and go test (TUG) and a significant increase in the berg balance scale (BBS) after treatment in experimental group (TUG: F_1.02, 7.4_ = 10.53, p = 0.01; BBS: F _1.05, 8.47_ = 81.30, p < 0.001), and control group (TUG: F _1.01, 8.11_ = 6.28, p = 0.03; BBS: F_1.03, 6.17_ = 19.16, p = 0.004). Pairwise comparisons showed a significant reduction in TUG at post-test and follow up in experimental group (p < 0.05). While, BBS score was significantly greater at one day and two weeks after treatment compared to the baseline in both groups (P < 0.05) (Table 2). Moreover, both variables significantly improved in post-test and follow-up test in experimental group compared to the control group (P < 0.05) (Table 2).

**Table 2:** Results of the balance assessment within time in each group and between groups

Parameters		Experimental (n = 10)		Control (n = 10)		
		Mean change score (SD)	p value ^a^	Mean change score (SD)	p value ^a^	p value between groups ^b^
BBS (score)	Pre-post	-10.66 ± 1.20	0.<001*	-3.57 ± 0.71	0.008*	0 < 001**
	Pre-follow	-11 ± 1.17	0.<001*	-4.14 ± 0.98	0.01*	0.002**
TUG (s)	Pre-post	36.60 ± 11.40	0.04*	22.01 ± 4.97	0.007*	0.03**
	Pre-follow	35.65 ± 10.75	0.03*	29.10 ± 11.99	0.12	0.03**
Overall-static (score)	Pre-post	1.82 ± 0.36	0.007*	1.57 ± 0.45	0.02*	0.04**
	Pre-follow	1.92 ± 0.43	0.01*	1.27 ± 0.53	0.13	0.36
Overall- dynamic (score)	Pre-post	2.01 ± 0.59	0.04*	0.96 ± 0.34	0.06	0.82
	Pre-follow	2.05 ± 0.60	0.04*	0.86 ± 0.37	0.14	0.13
AP static (score)	Pre-post	0.84 ± 0.19	0.008*	0.54 ± 0.35	0.52	0.04**
	Pre-follow	0.80 ± 0.27	0.06	0.37 ± 0.40	> 0.99	0.13
AP dynamic (score)	Pre-post	0.58 ± 0.12	0.003*	-0.03 ± 0.10	0.98	0.04**
	Pre-follow	0.50 ± 0.14	0.02*	-0.05 ± 0.12	0.87	0.41
ML static (score)	Pre-post	1.04 ± 0.33	0.04*	0.48 ± 0.32	0.56	0.42
	Pre-follow	1,18 ± 0.43	0.07	0.30 ± 0.42	0.97	0.17
ML dynamic (score)	Pre-post	0.72 ± 0.22	0.03*	0.24 ± 0.25	> 0.99	0.003**
	Pre-follow	0.72 ± 0.20	0.02*	0.34 ± 0.42	> 0.99	0.08

Values are expressed as mean ± standard deviation

Abbreviation: BSS, Berg Balance Scale; TUG, Time up & go test; AP, Antroposterior; ML, Medio Lateral; SD, Standard deviation

*Significance *p* < 0.05, a: Repeated measure ANOVA with Bonferroni post hoc (within group)

**Significance *p* < 0.05, b: Mann-Whitney U-test (between groups)

According to repeated measures ANOVA, significant reduction were observed after treatment in the postural stability indices for experimental group (overall static: F_2, 12_ = 21.11, p = 0.003; overall dynamic: F_1.09, 6.54_ = 11.13, p = 0.01; AP static: F_1.08, 8.65_ = 10.83, p = 0.009; AP dynamic: F_1.14, 9.13_ = 16.13, p = 0.002; ML static: F_1.02, 8.16_ = 8.11, p = 0.02; ML dynamic: F_1.12, 9.02_ = 11.09, p = 0.008), and for control group (overall static: F_1.08, 8.69_ = 8.18, p = 0.01; overall dynamic: F_1.12, 8.99_ = 6.29, p = 0.03). Furthermore, the experimental group showed significantly reduction in pairwise comparisons of all the variables in the post-test and follow-up test compared to the baseline (P < 0.05). No significant differences were observed in the control group in the post-test and follow-up test (P > 0.05) (Table 2). Only overall static, AP static, AP dynamic, and ML dynamic in post-test were significantly lower in the experimental group than the control group (P < 0.05) (Table 2).

**Fig. 2:**
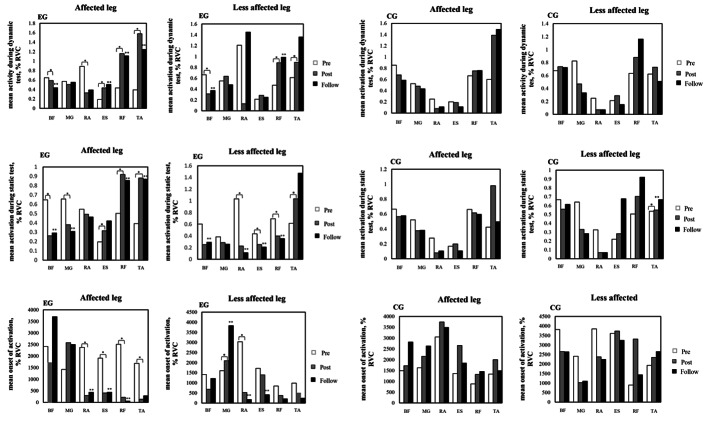
EMG activation during dynamic and static balance test before, one day and two weeks after intervention in each group. The figure on the left side indicates the mean activation of the affected sided muscles in each group. The figure on the right indicates the mean activation of the less- affected side in each group. BF – biceps femoris; MG – medial gastrocnemius; RF – rectus femoris; TA – tibialis anterior; RA – rectus femoris; ES – erector spine. ^*^ and ٭٭ show significant differences in muscle activation and onset time between pre-post and pre-follow up interventions, respectively (P < 0.05)

During the static balance test, repeated measures ANOVA detected a significant increase in the muscle activation of the affected side (rectus femoris: F_2, 16_ = 17.89, p < 0.001; tibialis anterior: F_1.07, 8.57_ = 13.36, P = 0.005), and a significant reduction in the erector spine (F_1.16, 9.29_ = 4.47, p = 0.02) in the experimental group. Also, the muscle activity of the less affected side (rectus femoris: F_1.01, 8.09_, P = 0.01; rectus abdominus: F_1, 8_ = 9.33, p = 0.01; erector spine: F_1, 8.02_ = 7.76, P = 0.02) significantly decreased in the experimental group. According to repeated measure ANOVA, no significant change was seen in the control group (P > 0.05) (Fig. 2).

Pairwise comparison showed that the activation of the rectus femoris, and tibialis anterior of the affected side increased and the biceps femoris and medial gastrocnemius decreased significantly in the experimental group (P < 0.05). Also, the activity was significantly lower in the biceps femoris, rectus abdominus, rectus femoris, and higher in the tibialis anterior of the less affected side in the experimental group (P < 0.05). No significant difference was observed in the control group (p > 0.05). Between-group differences showed significant changes in the activation of the biceps femoris of both sides in post- and follow up test, the rectus femoris of the affected side in post-test, erector spine of the affected side in follow-up test, and tibialis anterior of the less affected side in both post- and follow-up test (p < 0.05) (Table 3).

**Table 3:** Results of muscle activation during static test within time in each group and between groups

Parameters			Experimental (n = 10)		Control (n = 10)		
			Mean change score (SD)	p value ^a^	Mean change score (SD)	p value ^a^	p value between groups ^b^
Pre-Post test	BF	A	0.39 ± 0.07 0.31 ± 0.10	0.002* 0.05*	0.13 ± 0.20 0.10 ± 0.11	> 0.99	< 0.001**
		L				> 0.99	0.001**
	MG	A	0.21 ± 0.05 0.13 ± 0.08	0.01* 0.54	0.22 ± 0.15 0.47 ± 0.44	0.53	0.88
		L				0.96	0.29
	RF	A	-0.43 ± 0.09 0.29 ± 0.09	0.005* 0.03*	026 ± 0.09 -0.30 ± 0.15	0.11	0.007**
		L				0.29	0.09
	TA	A	-0.52 ± 0.13 -01.66 ± 0.54	0.01* 0.04*	-0.74 ± 0.60 0.03 ± 0.23	0.78	0.19
		L				0.94	0.001**
	RA	A	-0.08 ± 0.05 1.99 ± 0.65	0.41 0.04*	0.27 ± 0.31 0.40 ± 0.24	0.98	0.36
		L				0.45	0.76
	ES	A	-0.14 ± 0.03 0.19 ± 0.06	0.007* 0.07	0.010 ± 0.02 0.03 ± 0.05	> 0.99	0.17
		L				> 0.99	0.98
Pre-Follow test	BF	A	0.36 ± 0.07 0.27 ± 0.08	0.004* 0.03*	0.15 ± 0.24 0.13 ± 0.15	0.88	0.02**
		L				0.95	0.004**
	MG	A	0.23 ± 0.05 0.09 ± 0.09	0.01* 0.84	0.22 ± 0.16 0.49 ± 0.44	0.68	0.49
		L				0.90	0.36
	RF	A	-0.34 ± 0.08 0.31 ± 0.09	0.01* 0.03*	0.24 ± 0.09 -0.41 ± 0.24	0.10	0.08
		L				0.42	0.15
	TA	A	-0.46 ± 0.13 -01.66 ± 0.55	0.02* 0.053	0.009 ± 0.10 0.04 ± 0.31	> 0.99	0.12
		L				> 0.99	0.03**
	RA	A	-0.10 ± 0.05 1.99 ± 0.65	0.32 0.04*	0.26 ± 0.31 0.40 ± 0.24	0.98	0.42
		L				0.44	0.56
	ES	A	-0.22 ± 0.08 0.19 ± 0.07	0.08 0.07	0.012 ± 0.02 0.04 ± 0.05	> 0.99	0.003**
		L				> 0.99	0.63

Values are expressed as Mean (SD) of difference between two evaluation times. The minus sign next to the mean values means increase in muscle activation

Abbreviation: BF, biceps femoris; MG, medial gastrocnemius; RF, rectus femoris; TA, tibialis anterior; RA, rectus femoris; ES, erector spine; A, Affected side; L, Less affected side; SD, Standard deviation

*Significance *p* < 0.05, a: Repeated measure ANOVA with Bonferroni post hoc (within group)

**Significance *p* < 0.05, b: Mann-Whitney U-test (between groups)

During dynamic balance test, repeated measures ANOVA disclosed a significant increase of the affected side muscles (the erector spine: F_1.09, 8.74_ = 15.48, p < 0.001; rectus femoris: F_2, 16_ = 34.47, p < 0.001; tibialis anterior: F_2, 16_ = 23.82, p < 0.001), and a significant reduction of the biceps femoris (F_2, 16_ = 17.47, p < 0.001) in the experimental group. Also, a significant increase in the less affected side muscles (rectus femoris: F_2, 16_ = 15.54, p < 0.001; tibialis anterior: F_1.19, 9.51_= 7.24, p = 0.006), and a significant decrease in the biceps femoris (F_1.14, 9.16_ = 15.05, p < 0.001) were found in the experimental group. According to repeated measures ANOVA, no differences were detected in the control group (P > 0.05) (Fig. 2).

Pairwise comparison revealed a significant increase in the erector spine, rectus femoris, and tibialis anterior of the affected side and a significant decrease of the biceps femoris in the experimental group (P < 0.05). There was also a significant increase in the activation of the rectus femoris, and tibialis anterior of the less affected side, and a significant decrease in biceps femoris in the experimental group (P < 0.05). Pairwise comparison showed no difference in the control group (p > 0.05) (Table 4). Moreover, the biceps femoris of both sides in post-test, the biceps femoris of the less affected side in follow-up test, and the erector spine of the affected side in both post- and follow-up test changed significantly between the groups (P < 0.05) (Table 4).

**Table 4:** Results of muscle activation during dynamic test within time in each group and between groups

Parameters			Experimental (n = 10)		Control (n = 10)		
			Mean change score (SD)	p value ^a^	Mean change score (SD)	p value ^a^	p value between groups ^b^
Pre-Post test	BF	A	0.43 ± 0.08 0.35 ± 0.07	0.002* 0.003*	0.25 ± 0.35 -0.06 ± 0.17	> 0.99 > 0.99	0.007**
		L					0.004**
	MG	A	0.01 ± 0.09 -0.07 ± 0.11	0.98 0.96	0.11 ± 0.19 0.59 ± 0.74	0.98 0.94	0.94
		L					0.29
	RF	A	-0.67 ± 0.09 -0.40 ± 0.09	< 0.001* 0.007*	-0.14 ± 0.14 -0.34 ± 0.18	> 0.99 0.31	0.07
		L					0.65
	TA	A	-1.27 ± 0.18 -0.46 ± 0.10	< 0.001* 0.006*	-1.07 ± 0.68 -0.10 ± 0.22	0.50 > 0.99	0.15
		L					0.19
	RA	A	0.25 ± 0.10 1.05 ± 0.65	0.12 0.43	0.23 ± 0.27 0.25 ± 0.20	> 0.99 0.78	0.36
		L					0.65
	ES	A	-0.28 ± 0.07 -0.04 ± 0.08	0.01* 0.08	0.05 ± 0.03 -0.009 ± 0.07	0.44 > 0.99	0.007**
		L					0.94
Pre-Follow test	BF	A	0.34 ± 0.08 0.31 ± 0.09	0.008* 0.03*	0.44 ± 0.35 0.10 ± 0.25	0.75 > 0.99	0.06
		L					0.03**
	MG	A	0.002 ± 0.07 0.08 ± 0.15	0.94 0.98	0.17 ± 0.16 0.69 ± 0.72	0.93 0.98	0.15
		L					0.36
	RF	A	-0.65 ± 0.10 -0.51 ± 0.09	< 0.001* 0.003*	0.007 ± 0.07 -0.47 ± 0.31	> 0.99 0.53	0.12
		L					0.63
	TA	A	-0.87 ± 0.14 -0.80 ± 0.27	0.001* 0.05	-0.75 ± 0.59 0.16 ± 0.12	0.75 0.62	0.95
		L					0.10
	RA	A	0.22 ± 0.21 1.04 ± 0.75	> 0.99 0.61	0.22 ± 0.27 0.25 ± 0.20	0.95 0.77	0.87
		L					0.63
	ES	A	-0.31 ± 0.08 -0.07 ± 0.07	0.01* > 0.99	0.02 ± 0.03 0.01 ± 0.06	> 0.99 > 0.99	0.004**
		L					0.12

Values are expressed as Mean (SD) of difference between two evaluation times. The minus sign next to the mean values means increase in onset time

Abbreviation: BF, biceps femoris; MG, medial gastrocnemius; RF, rectus femoris; TA, tibialis anterior; RA, rectus femoris; ES, erector spine; A, Affected side; L, Less affected side; SD, Standard deviation

*Significance *p* < 0.05, a: Repeated measure ANOVA with Bonferroni post hoc (within group)

**Significance *p* < 0.05, b: Mann-Whitney U-test (between groups)

According to repeated measures ANOVA, the onset time reduced significantly in the affected side muscles (rectus abdominus: F_2, 16_ = 11.51, p < 0.001; erector spine: F_2, 16_ = 8.38, p = 0.003; rectus femoris: F_2, 16_ = 28.122, p < 0.001; tibialis anterior: F_2, 16_ = 7.90, p = 0.004), and the less affected side muscles (rectus abdominus: F_1.2, 9.61_ = 9.96, p = 0.009; erector spine: F_2, 16_ =5.17, p = 0.01; medial gastrocnemius: F_2, 16_ = 7.67, p = 0.005) in the experimental group. Repeated measures ANOVA showed no significant change in the onset time of the muscles in the control group (Fig. 2). The onset times for all muscles of the experimental group except the medial gastrocnemius of both sides and the biceps femoris of the affected side were significantly less than those of the control group in the post-test (p < 0.05) (Fig. 2).

Pairwise comparison showed a significant reduction in the onset time of the rectus abdominus, erector spine, rectus femoris, and tibialis anterior muscles of the affected side in the experimental group (p < 0.05). Also, in the experimental group, the onset time significantly reduced in the rectus abdominus and erector spine of the less affected side significantly and increased in the medial gastrocnemius (P < 0.05). However, no significant change was observed in the control group (p > 0.05). Significant differences were found in the rectus abdominus, tibialis anterior, and rectus femoris of the both side, biceps femoris and medial gastrocnemius of the less affected side and the erector spine of the affected side in post-test between groups. Also, the rectus femoris, rectus abdominus, and erector spine of the both sides and the biceps femoris, medial gastrocnemius, and tibialis anterior of the less affected side changed significantly in the experimental group compared to the control group (p < 0.05) (Table 5).

**Table 5:** Results of onset time within time in each group and between groups

Parameters			Experimental (n = 10)		Control (n = 10)		
			Mean change score (SD)	p value ^a^	Mean change score (SD)	p value ^a^	p value between groups ^b^
Pre-Post test	BF	A	780.55 ± 1172.47 700.66 ± 911.35	0.87 0.93	-917.00 ± 1014.54 -179.57 ± 466.93	0.97 0.94	0.94
		L					0.01**
	MG	A	-1474.44 ± 685.68 -1222.00 ± 254.81	0.19 0.004*	-448.85 ± 377.84 2174.00 ± 1372.36	0.83 0.49	0.59
		L					0.01**
	RF	A	2507.44 ± 400.02 472.77 ± 1056.30	< 0.001* > 0.99	-330.42 ± 385.18 -224.00 ± 180.15	> 0.99 0.78	< 0.001**
		L					0.01**
	TA	A	1601.55 ± 295.79 548.55 ± 359.96	0.002* 0.49	-945.85 ± 474.98 -540.71 ± 608.68	0.28 > 0.99	< 0.001**
		L					0.01**
	RA	A	2112.00 ± 445.14 2789.77 ± 694.06	0.004* 0.01*	-1225.71 ± 748.91 128.28 ± 387.33	0.45 > 0.99	0.003**
		L					0.001**
	ES	A	1733.00 ± 369.10 274.11 ± 609.01	0.005* > 0.99	-275.28 ± 373.47 -116.14 ± 293.67	> 0.99 > 0.99	< 0.001**
		L					0.07
Pre-Follow test	BF	A	-1013.00 ± 1205.21 341.00 ± 385.57	0.93 0.97	-1327.14 ± 1293.56 110.71 ± 515.51	0.94 0.98	0.63
		L					0.01**
	MG	A	-1008.22 ± 786.70 -1896.66 ± 576.25	0.70 0.03*	-141.71 ± 497.47 1810.14 ± 839.04	> 0.99 0.22	0.79
		L					0.01**
	RF	A	2689.44 ± 471.32 1194.55 ± 698.83	0.001* 0.37	-362.71 ± 315.23 -309.85 ± 576.65	0.881 > 0.99	0.01**
		L					0.003**
	TA	A	1459.44 ± 555.16 900.33 ± 506.56	0.09 0.34	100.14 ± 664.88 -368.00 ± 571.23	> 0.99 > 0.99	0.25
		L					0.03**
	RA	A	2014.77 ± 581.44 3189.11 ± 1040.36	0.02* 0.04*	-1352.42 ± 790.13 -89.00 ± 440.87	0.41 0.97	0.01**
		L					0.01**
	ES	A	1740.00 ± 566.27 1523.00 ± 353.03	0.04* 0.008*	-150.00 ± 432.37 53.28 ± 516.18	0.95 > 0.99	0.03**
		L					0.01**

Values are expressed as Mean (SD) of difference between two evaluation times. The minus sign next to the mean values means increase in onset time

Abbreviation: BF, biceps femoris; MG, medial gastrocnemius; RF, rectus femoris; TA, tibialis anterior; RA, rectus femoris; ES, erector spine; A, Affected side; L, Less affected side; SD, Standard deviation

*Significance *p* < 0.05, a: Repeated measure ANOVA with Bonferroni post hoc (within group)

**Significance *p* < 0.05, b: Mann-Whitney U-test (between groups)

## Discussion

This study aimed to determine whether the hip joint mobilization with movement technique affected the EMG muscle activity, postural control, and functional and dynamic balance among chronic post-stroke patients. The between-group analyses revealed some significant improvements in most of the variables in the experimental group compared to the control group.

Hip joint mobilization with movement technique plays important roles in repositioning the joint and normalizing tracking [29]. Moreover, the main focus of post-stroke rehabilitation is on selective and isolative joint movements to promote balance ability [30, 31]. Proximal joint kinematics compensates the distal limb deficits and consequently affects the ankle joint. Therefore, the level of lower extremity motor control and proximal lower extremity selective motor control is of great importance compared to distal lower extremity control [30, 31].

This study indicated a significant improvement in both timed up and go test and berg balance scale in both groups, which is consistent with previous studies on the effect of mobilization with movement technique on balance [32–34]. Mobilization with movement technique normalizes accessory movements by increasing the flexibility of non-contractile tissues even in primary neurological pathology [35].

The results of the current study indicated the postural stability variables significantly reduced after treatment in the experimental group, which is in agreement with a previous study in which talocrural mobilization with movement technique combined with conventional physiotherapy modified ankle kinetics and balance in post-stroke patients [36].

Among the multiple hypotheses related to the effectiveness of the mobilization with movement technique, both peripheral (positional fault) and central (neurophysiology) mechanisms have been discussed [37–41]. The lateral gliding during hip joint mobilization with movement technique affects local mechanoreceptors and creates reflexological inhibition, which modifies local arthrokinematics and optimizes load distribution on damaged tissues [37–39, 41–45].

In the experimental group, the amplitude of extensor muscles (rectus femoris, tibialis anterior, and erector spine) increased and the onset time decreased on the affected side during static balance test, accompanied by decreased amplitude and onset time of the extensors on the other side. Meanwhile, during the dynamic test, the EMG activity increased and the onset time decreased on both sides. Post-stroke patients rely mostly on the less affected side to compensate both the delayed and weak muscle responses on the affected side [30].

The results indicated equal reliance on both sides during the static balance test due to reduced activation of knee, ankle, and hip muscles. The increased activity on both sides during dynamic balance test may indicate bilateral muscle coordination against larger disturbances. Moreover, hip joint mobilization with movement technique can cause cross-activation on the contralateral untrained side. Also, the greater activity of rectus femoris and tibialis anterior muscles were recorded after hip joint mobilization with movement technique through proximal lower limb selective motor control and the kinematic chain from proximal to distal joints.

The impaird muscle activity preparation on the affected side along with the inability to produce the maximum sustained effort are some possible reasons for greater variability in the sequence of muscle activity and postural instability [46]. In post-stroke patients, a delayed onset is present in a normal pattern of distal-proximal agonist muscle activation or a replacement in the sequence of agonist-antagonist co-contraction activity [47]. Moreover, the postural adjustment to external disturbances is defined by the delayed onset time and a low amplitude of lower extremity muscles [48, 49]. Since there were no significant differences at baseline among groups, the recovery of muscle activity in the experimental group compared with control group can be attributed to the Mulligan’s principle.

Delayed trunk muscle reaction, trunk muscle weakness, and impairment of trunk position sense and performance are common in post-stroke patients [50–52]. Trunk postural control plays a greater role in proximal stability for distal lower extremity activity [53]. Impaired trunk postural control results from impaired control of trunk muscles voluntary contraction. Our findings are in line with those of previous studies reporting that the coordinated activity of lower extremity and trunk muscles with adequate timing and amplitude is required to maintain standing balance [51].

This study had some limitations. Firstly, the results may not be generalized to acute or sub-acute stroke patients. In this study, patients had chronic stroke and cannot represent all post-stroke patients. Secondly, the participants in the experimental group might have a better result regardless of the extra time in each treatment session. The comparison between the experimental group and the placebo-control or the sham-control group is needed to elucidate these effects. Finally, this study included a small sample size, and therefore small sample size may make it difficult to generalize the statistical results. Further studies are required to evaluate the efficacy of hip joint mobilization with movement technique combined with conventional physiotherapy at all stages of stroke and compare the proximal and distal joints.

## Conclusion

Hip joint mobilization with movement technique combined with conventional physiotherapy improved the muscle activity, postural stability, and balance. Greater improvement was found in muscle activity and clinical outcomes in experimental group compared to conventional physiotherapy. These findings would help to design an appropriate and novel therapeutic plan for chronic post-stroke patients.

## Data Availability

The datasets used and/or analyzed during the current study available from the corresponding author on reasonable request.

## List of abbreviations

A: Affected side
AP: Antroposterior
BBS: Berg balance scale
BF: Biceps femoris
EMG: Electromyography
ES: Erector spine
IRCT: Iranian Registry of Clinical Trials
L: Less affected side
MG: Medial gastrocnemius
ML: Medio lateral
RA: Rectus abdominus
RF: Rectus femoris
ROM: Range of motion
SD: Standard deviation
TA: Tibialis anterior
TUG: Time up and go test
